# Powerful bacterial killing by buckwheat honeys is concentration-dependent, involves complete DNA degradation and requires hydrogen peroxide

**DOI:** 10.3389/fmicb.2012.00242

**Published:** 2012-07-04

**Authors:** Katrina Brudzynski, Kamal Abubaker, Tony Wang

**Affiliations:** ^1^API-Medicals, Brock University, St. CatharinesON, Canada; ^2^Department of Biological Sciences, Brock University, St. CatharinesON, Canada

**Keywords:** bactericidal action, honey, MBC/MIC ratio, killing curves, MRSA and VRE, hydrogen peroxide, hydroxyl radicals, aminophenyl fluorescein

## Abstract

Exposure of bacterial cells to honey inhibits their growth and may cause cell death. Our previous studies showed a cause-effect relationship between hydroxyl radical generated from honey hydrogen peroxide and growth arrest. Here we explored the role of hydroxyl radicals as inducers of bacterial cells death. The bactericidal effect of ·OH on antibiotic-resistant clinical isolates of MRSA and VRE and standard bacterial strains of *E. coli* and *B. subtiles* was examined using a broth microdilution assay supplemented with 3′-(*p*-aminophenyl) fluorescein (APF) as the ·OH trap, followed by colony enumeration. Bactericidal activities of eight honeys (six varieties of buckwheat, blueberry and manuka honeys) were analyzed. The MBC/MIC ratio ≤4 and the killing curves indicated that honeys exhibited powerful, concentration-dependent bactericidal effect. The extent of killing depended on the ratio of honey concentration to bacterial load, indicating that honey dose was critical for its bactericidal efficacy. The killing rate and potency varied between honeys and ranged from over a 6-log_10_ to 4-log_10_ CFU/ml reduction of viable cells, equivalent to complete bacterial eradication. The maximal killing was associated with the extensive degradation of bacterial DNA. Honey concentration at which DNA degradation occurred correlated with cell death observed in the concentration-dependent cell-kill on agar plates. There was no quantitative relationship between the ·OH generation by honey and bactericidal effect. At the MBC, where there was no surviving cells and no DNA was visible on agarose gels, the ·OH levels were on average 2–3x lower than at Minimum Inhibitory Concentration (MICs) (*p* < 0.0001). Pre-treatment of honey with catalase, abolished the bactericidal effect. This raised possibilities that either the abrupt killing prevented accumulation of ·OH (dead cells did not generate ·OH) or that DNA degradation and killing is the actual footprint of ·OH action. In conclusion, honeys of buckwheat origin exhibited powerful, concentration-dependent bactericidal effect. The killing and DNA degradation showed a cause-effect relationship. Hydrogen peroxide was an active part of honey killing mechanism.

## Introduction

At present, the exact mechanism of honey action that leads to bacterial cell death is unknown. The early honey research was focused primarily on providing evidence of antibacterial activity by defining the spectrum of susceptible bacteria, by comparing efficacy of different honeys in bacterial growth inhibition and by identifying the activity-related compounds (Allen et al., 1991; Molan, 1992; Cooper et al., 1999, 2000, 2002a,b; Lusby et al., 2002; Wilkinson and Cavanagh, 2005; Brudzynski and Kim, 2011). In due course, it became apparent that there are two main groups of honeys with the respect to the main component involved in bacterial growth inhibition: the group of European and American honeys whose activity was catalase-sensitive and showed substantial correlation with the internal levels of hydrogen peroxide and the group of honeys of *Leptospermum* spp. whose activity was hydrogen peroxide-independent (Molan and Russell, 1988; Allen et al., 1991) but instead correlated well with the levels of internal methylglyoxal (Adams et al., 2008; Mavric et al., 2008). Bacterial cultures exposed to the former group of honeys showed signs of increased oxidative stress that correlated with generation and accumulation of hydroxyl radicals (Brudzynski et al., 2011, 2012; Brudzynski and Lannigan, 2012). Our recent studies documented that hydrogen peroxide was a necessary substrate for ·OH formation via the metal-catalyzed Fenton reaction. ·OH formation and accumulation inhibited bacterial growth in a dose-dependent manner. Addition of transition metal, Cu(II) to this system enhanced honey bacteriostatic action as manifested by a marked decrease of Minimum Inhibitory Concentration MIC values against both standard and antibiotic-resistant clinical isolates (Brudzynski et al., 2011; Brudzynski and Lannigan, 2012). These studies provided evidence that ·OH generated from honey H_2_O_2_ occupied a key position in the bacteriostatic mechanism of action.

It was therefore plausible that ·OH radicals may play similar role in the bactericidal effect of honey. ·OH radicals are powerful but short-lived oxidants that indiscriminately target macromolecules located in close vicinity to sites of ·OH formation (Roots and Okada, 1975). In bacterial cells, ·OH radicals were shown to cause protein and lipid peroxidation, and DNA and RNA degradation. The oxidative injury to these macromolecules impaired permeability of cell membranes and cell proliferation, respectively, and ultimately led to the decrease in cell viability and cell death (Imlay and Linn, 1988; Imlay et al., 1988; Cabiscol et al., 2000; Sakihama et al., 2002). Recently, Kohanski et al. (2007, 2010) provided evidence that bactericidal efficacy of different groups of antibiotics was ultimately linked to the overproduction of hydroxyl radicals inside bacterial cell. These results gave support to our hypothesis that ·OH produced from honey's hydrogen peroxide may also underlie the bactericidal action of honey.

The bactericidal effect of antimicrobial drugs is usually characterized by pharmacodynamic parameters. However, the dynamics of bacterial killing by different honeys have not been thoroughly investigated. Honey antibacterial activity is commonly defined in terms of its growth inhibitory activity and usually quantitated using the MIC method. Quite often in literature this activity was equated with honey ability to kill microorganisms. In only a couple of examples have data from bacteriostatic and bactericidal assays been simultaneously analyzed (Blair et al., 2009; Tan et al., 2009; Sherlock et al., 2010). A recurrent finding from the above studies was that the maximal growth inhibitory and bactericidal effects of honeys lied in the narrow concentration range. The MICs for honeys of different botanical origins ranged between 4–16% w/v, averaging around 8% w/v (Willix et al., 1992; Cooper and Molan, 1999; Cooper et al., 1999, 2000; Blair et al., 2009; Brudzynski et al., 2011). Clearly, concentrations of honey active components were critical for the antibacterial effects and this information gave the first indication of a possible mode of bactericidal action.

Recent results from our laboratory have suggested that the oxidative stress evoked by honeys on bacterial cells resulted from the coupling chemistry between polyphenols, H_2_O_2_ and transition metals. Concentrations of these components were responsible for the suppression of bacterial growth (MIC) as well as for the extent of DNA degradation (Brudzynski et al., 2012). The single and double DNA strand-breaks have been clearly observed after incubation of plasmid DNA with honeys of different botanical origin. DNA degrading potencies of these honeys were closely related to the total phenolic content and redox capacity of polyphenols (Brudzynski et al., 2012). The latter activity play central role in facilitating the polyphenol-mediated Fenton reaction and generation of hydroxyl radicals (Sakihama et al., 2002). Depending on the concentration and in the presence of catalytic amounts of transition metals, flavonoids of certain structure induce free radicals generation (Cao et al., 1997; Fukumoto and Mazza, 2000).

Indeed, polyphenols emerged as important functional honey constituents. The redox capacity of polyphenols enabled them to interact with each other, and with proteins and sugars leading to a formation of high molecular structures called melanoidins (Brudzynski and Miotto, 2011a,b). In the structure-depending way, polyphenols were capable of influencing the levels of antioxidant and antibacterial activities as well as the extent of polymerization of active honey components (Brudzynski and Miotto, 2011a,b,c). As a consequence of their redox activity, the balance between antioxidant/proxidant activities of polyphenols could be changed in the presence of oxygen or hydrogen peroxide and traces of metal ions, and result in the generation of cytotoxic free radicals (Cao et al., 1997; Fukumoto and Mazza, 2000; Sakihama et al., 2002). These in turn, could be responsible for cell injury and DNA damage, mimicking the antibacterial action of phagocytic cells (Gutteridge et al., 1998) or antibiotics (Kohanski et al., 2007, 2010). The concentration of polyphenols and hydrogen peroxide in different honeys may therefore be of critical importance for bacterial cell survival.

The aim of the present study was to (a) demonstrate and compare bactericidal effect of different honeys, (b) establish pharmacodynamic parameters such as killing rates, maximum bactericidal concentration and potency of different honeys, and (c) to explore the role of hydroxyl radical in the killing mechanism of honey.

## Materials and methods

### Honeys

Honeys were donated by Canadian beekeepers and included both commercial (pasteurized) and apiary (unprocessed) samples. The list of honeys and their plant origin is given in Table 1.

**Table 1:** **List of honeys**.

**Honey**	**Botanical source**	**Color (A_560_–A_720_)**	Hydrogen peroxide (mM/L)^*^
Spl. 15	*Vaccinium corymbosum* Blueberry	0.267	1.75 ± 0.02
Spl. 23	*Fagopyrum esculentum* Buckwheat	0.975	2.12 ± 0.022
Spl. 77	*Fagopyrum esculentum* Buckwheat	1.266	2.70 ± 0.06
Manuka	*Leptospermum scoparium*	0.539	1.04 ± 0.17
H203	*Fagopyrum esculentum* Buckwheat	0.300	0.248 ± 0.020
H204	*Fagopyrum esculentum* Buckwheat	0.320	0.740 ± 0.08
H205	*Fagopyrum esculentum* Buckwheat	0.463	1.17 ± 0.05
H206	*Fagopyrum esculentum* Buckwheat	0.965	1.11 ± 0.02

### Bacterial strains

Standard strains of *Bacillus subtilis* (ATCC 6633) and *Escherichia coli* (ATCC 14948) purchased from Thermo Fisher Scientific Remel Products (Lenexa, KS 66215) were grown in Mueller-Hinton Broth (MHB) (Difco Laboratories) overnight in a shaking water bath at 37°C. Overnight cultures were diluted with broth to the equivalent of the 0.5 McFarland standard.

Clinical isolates, vancomycin-resistant *Enteroccus faecium* (VRE2) and methicillin-resistant *Staphylococcus aureus* (MRSA6) were obtained from the Clinical Microbiology Laboratory of the London Health Science Centre, London Ontario. Strains were subcultured from swabs onto Mueller-Hinton II agar (Difco Laboratories). Isolates were identified to genus and species and their susceptibility to antibiotics was confirmed using an automated system (Vitek^R^, Biomérieux^R^). The presence of the *mec*A gene, *nuc* genes and *van*A and B genes were determined by polymerase chain reaction. This work was conducted by the Clinical Microbiology Laboratory, London Health Sciences Centre, London, Ontario as described previously (Brudzynski and Lannigan, 2012).

### Broth microdilution assay

The antibacterial activity of honeys was performed using a broth microdilution assay in sterile, 96-well format as described previously (Brudzynski et al., 2011). Bacterial growth was measured at A_595_ nm using the Synergy HT multidetection microplate reader (Synergy HT, Bio-Tek Instruments, Winooski, VT, USA). In a single experiment, each honey was tested in triplicate. Each microorganism has been analyzed at least in three independent experiments.

Statistical analysis and dose response curves were obtained using K4 software provided by Synergy HT (Bio-Tek Instruments, Winooski, VT, USA).

The MIC were determined from the growth inhibition profiles curves and represented the lowest concentration of the honeys that inhibited the bacterial growth by 90% as measured by the absorbance at A_595_ nm.

### Killing curves and determination of MBC

After determination of the MIC for each strain in broth microdilution assay, the killing curves were constructed by subculturing the entire contents of each well (100 μl) from microplates that showed no visible growth onto Mueller-Hinton agar (MHA). The killing curves were produced from serially diluted honeys against each bacterial strain. To verify the final cell density of bacteria not exposed to honey, wells containing inoculum only (assay control) were serially 10-fold diluted with sterile water to obtain approximate cell density of 10^4^ and 10^2^ CFU/ml and then 10-μl and 100-μl aliquot from each dilution was streaked onto agar plates.

The MBC endpoint was the minimum concentration of honey at which at least 99.9% of the initial inoculum was eradicated and at which only one or no colonies could be seen on MHA.

### Hydroxy-radicals measurements

3′-(*p*-aminophenyl) fluorescein (APF) (Invitrogene, Canada) was used for the detection of hydroxyl radicals produced by honeys. A generation of ·OH radicals was monitored using a broth microdilution assay by adding 10 μM of APF (in 50 mM potassium phosphate buffer, pH 7.4) to each experimental wells (containing bacterial inoculum and honey dilutions) as well as the assay controls (bacterial inoculum). The negative control consisted of experimental wells without APF.

The plates were analyzed for both, bacterial growth using absorbance at A_595_ nm and hydroxyl radical generation using fluorescence excitation and emission wavelengths at 490 and 520 nm, respectively.

### Bacterial DNA isolation

Overnight *E. coli* cultures were adjusted to 10^7^ CFU/ml in M-H broth and incubated in 1:1 ratio with honeys at their 2xMBCs and MBCs by shaking (250 rpm) at 37°C overnight for 18 h. Cells were recovered by centrifugation at 3000 × g (Eppendorf) for 60 s The total genomic DNA was isolated from untreated and from honey-treated cells using two methods: plasmid DNA isolation kit (MiniPrep) to monitor DNA degradation fragments and bacterial genomic DNA isolation kit (Norgen Biotek Corporation, St. Catharines, ON., Canada) according to the manufacturer's instructions.

### Agrose gel electrophoresis

Ten microliter aliquots of isolated DNA were analyzed by agarose gel electrophoresis (1%) in 1X TAE buffer containing ethidium bromide with a Gel System from Bio-Rad Laboratories (Mississauga, Ontario). The DNA molecular weight marker was selected to be the HighRanger 1 kb DNA Ladder, MidRanger 1 kb DNA Ladder and PCRSizer 100 bp DNA Ladder from Norgen Biotek Corporation (St. Catharines, ON., Canada). The gels were run at 85 V for 40 min and then visualized and photographed using the Gel Doc 1000 system and the Quantity One 1-D Analysis software (version 4.6.2 Basic) from Bio-Rad.

### Statistical analysis

The GraphPad Instant software version 3.05 (GraphPad Software Inc.) was used to calculate the means and standard deviation in experiments involving triplicate and multiple replicate analyses of samples. Differences between averages were evaluated by the *t*-test with the significance level of *p* ≤ 0.05.

## Results

### Bactericidal effect of honeys

To examine bactericidal effects of honeys, the isolates of MRSA6 and VRE2 and standard bacteria, *E. coli* and *B. subtilis* were analyzed under the same growth conditions, bacterial density (10^7^CFU/ml), incubation times (18 h) and temperature (37°C). The MIC and MBC values were determined using broth microdilution assay with the endpoint at which 90% bacteria were inhibited or killed, respectively. The assay was followed by a colony enumeration in the standard plate count.

Among eight honeys tested, the MIC and MBC values ranged from 3.125% to 25% v/v, indicating a rather narrow concentration range at which honey was effective. There was no difference in median MIC and MBC values obtained in three separate experiments conducted in triplicate over the period of nine month. As shown in Table 2, the MBC/MIC ratio ≤4 indicated that the honeys displayed a predominantly bactericidal activity.

**Table 2:** **Comparison of bactericidal activity of honeys and bacterial susceptibility**.

**Honey variety**	**MRSA6**		**VRE2**		***E. coli***		***B. subtilis***	
	**MIC** (**%, v/v**)	**MBC** (**%, v/v**)	**MIC** (**%, v/v**)	**MBC** (**%, v/v**)	**MIC** (**%, v/v**)	**MBC** (**%, v/v**)	**MIC** (**%, v/v**)	**MBC** (**%, v/v**)
H15	25%	50%	25%	50%	50%	50%	50%	50%
H23	6.25%	12.5%	12.5%	25%	12.5%	25%	12.5%	12.5%
H77	3.125%	6.25%	12.5%	12.5%	12.5%	25%	12.5%	12.5%
M2	3.125%	6.25%	12.5%	25%	12.5%	12.5%	6.25%	6.25%
H203	6.25%	12.5%	25%	25%	25%	25%	12.5%	12.5%
H204	6.25%	12.5%	25%	25%	12.5%	12.5%	12.5%	12.5%
H205	6.25%	12.5%	25%	25%	12.5%	12.5%	12.5%	12.5%
H206	3.125%	6.25%	6.25%	12.5%	6.25%	6.25%	12.5%	12.5%

### Bacterial killing by honey occurred in a concentration-dependent manner

**Figure 1:**
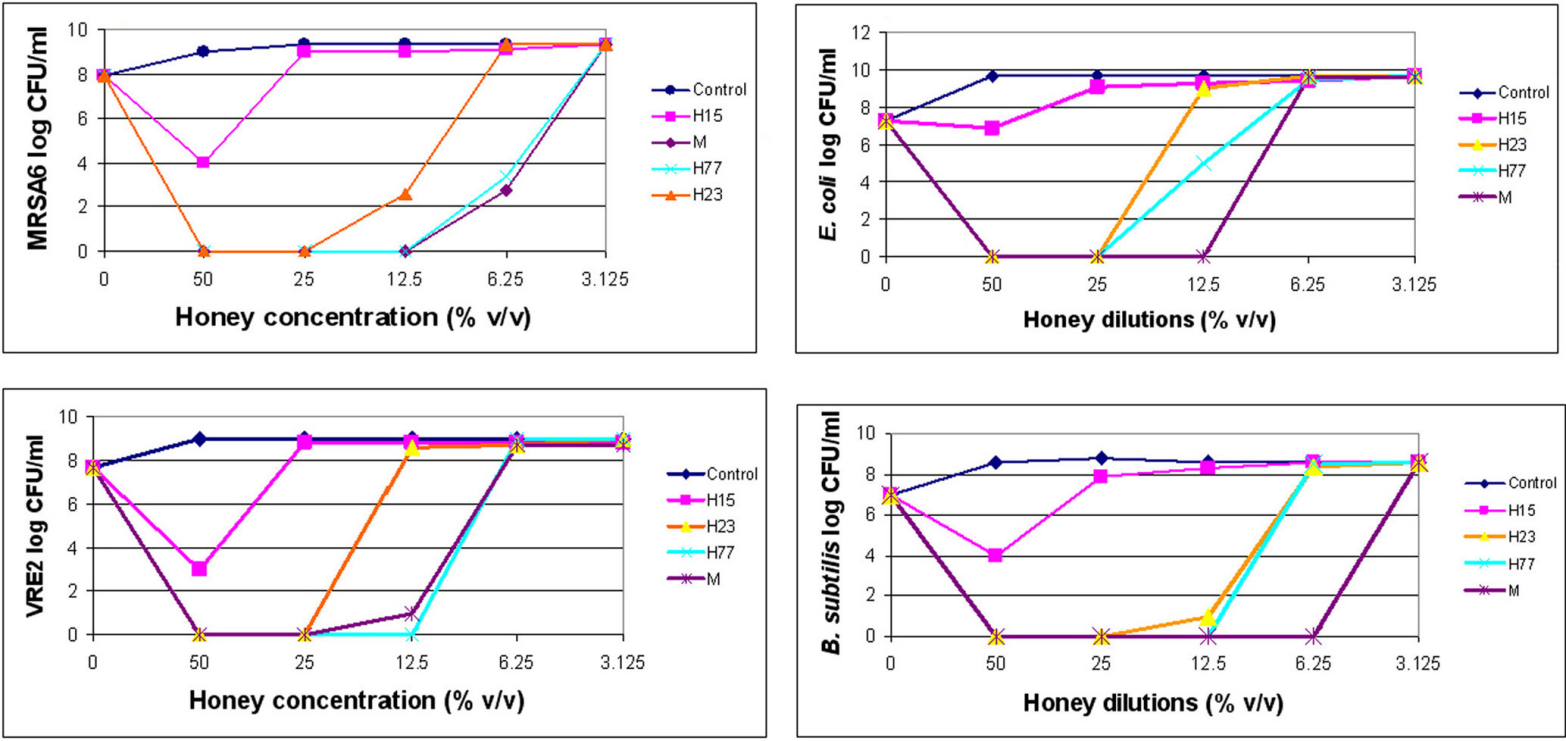
**Killing curves of honey for MRSA6, VRE2, *E. coli* and *B. subtilis*.** Honey bactericidal effects were evaluated at multiples of MIC. The figure displays concentration-dependent reduction in log CFU/ml and subsequent regrowth of bacteria for each honey.

Dynamics of bacterial killing was investigated as a function of honey concentrations. The changes in the bacterial cell count were assessed at 4×MIC, 2×MIC and the MIC. Killing curves showed a rapid and complete reduction of cell viability at or above the MBCs (Figure 1). After honey concentrations fall below MBC levels, the bactericidal effect was abruptly diminished and the bacterial re-growth emerged. Since honeys were bactericidal in the narrow zone of concentrations, it suggested that the killing critically depended on the ratio of honey concentration to bacterial load. It is predicted that with the increased bacterial cell densities, higher concentration of honey would be needed to produce the maximal killing effect.

Bactericidal potency, that is the range of concentrations over which honey produced killing effect, seemed to depend on honey variety. Blueberry honey required the highest concentration (50% v/v) to kill MRSA6 and VRE2 as well as *E. coli* and *B. subtilis*, while buckwheat honeys and manuka did kill bacteria but they differ in their MBC (H23< H77< manuka) (Table 2, Figure 1). Similarly, buckwheat honeys H77 and manuka showed higher bactericidal potency than buckwheat honey H23 because they evoked the same maximal bactericidal effect (a complete bacterial eradication) at lesser concentrations (12.5% v/v vs 25% v/v, respectively) (Figure 1).

Nevertheless, the killing curves demonstrated that honeys at their MBCs caused >6 log_10_ CFU/ml reduction of colony counts, equivalent to complete bacterial eradication.

### Bacterial killing by honeys correlated with a complete DNA degradation

The observed absence of viable colony counts on agar plates at the MBC endpoints could be indicative of the extensive, catastrophic damage to cell integrity (Figure 1). To gather more insight into the mechanism of bacterial cell death after exposure to honey, we investigated a potential causal link between cell killing and DNA damage. DNA degradation and release of DNA fragments was analyzed by agarose gel electrophoresis by employing two methods of DNA extraction, genomic and plasmid DNA isolation methods, respectively.

**Figure 2:**
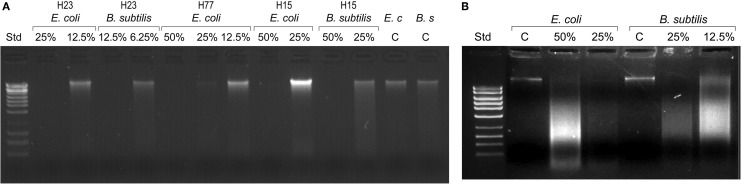
**Bacterial DNA degradation by honeys at bactericidal concentrations.** Cultures of *E. coli* and *B. subtilis* (10^7^ CFU/ml) were treated with honeys at concentrations ranging from 50% v/v to 6.25% v/v, equivalent to multiples of MBC for given honey. DNA degradation was analyzed on 1% agarose gels followed by DNA isolation using bacterial genomic DNA isolation method **(A)** and MiniPrep DNA isolation method **(B)**.

Since the bacterial killing occurred in the narrow concentration range, we examined whether honey concentrations played analogous role in DNA degradation. *E. coli* and *B. subtilis* cultures were exposed to honey H23 at 4×MIC, 2×MIC and MIC (50%, 25% and 12.5% v/v) and the integrity of their genomic DNA was analyzed on agarose gels. Honeys at their MBC caused extensive double-strand DNA cleavage that led to complete break down of genomic DNA (Figure 2A). The appearance of a smudge of small DNA fragments isolated from these cultures using plasmid DNA method captured ongoing DNA degradation (Figure 2B). Single strand cuts to the genomic DNA would not yield small fragments and thus would be undetectable using this method. This indicated that DNA degradation involved irreparable double-strand cuts which are lethal for the cell.

In contrast, honey at concentrations beyond the MBC did not degrade DNA. Thus, the complete DNA degradation appeared only at bactericidal concentration. Honey concentration at which DNA degradation occurred correlated well with cell death observed in the concentration-dependent cell-kill on agar plates (Figure 1).

These results indicated that bacterial killing and DNA degradation were interdependent events. Thus, there was a cause-and-effect relationship between honey MBCs, cell damage and DNA degradation.

### Bacterial killing by honey required hydrogen peroxide

Honey hydrogen peroxide played an essential role in the bacterial growth inhibition by being a substrate for hydroxyl radical generation (Brudzynski et al., 2011). Hydroxyl radicals inhibited in a dose-dependent manner the growth of several MRSA and VRE clinical isolates as well as standard *E. coli* and *B. subtilis* (Brudzynski et al., 2012).

**Figure 3:**
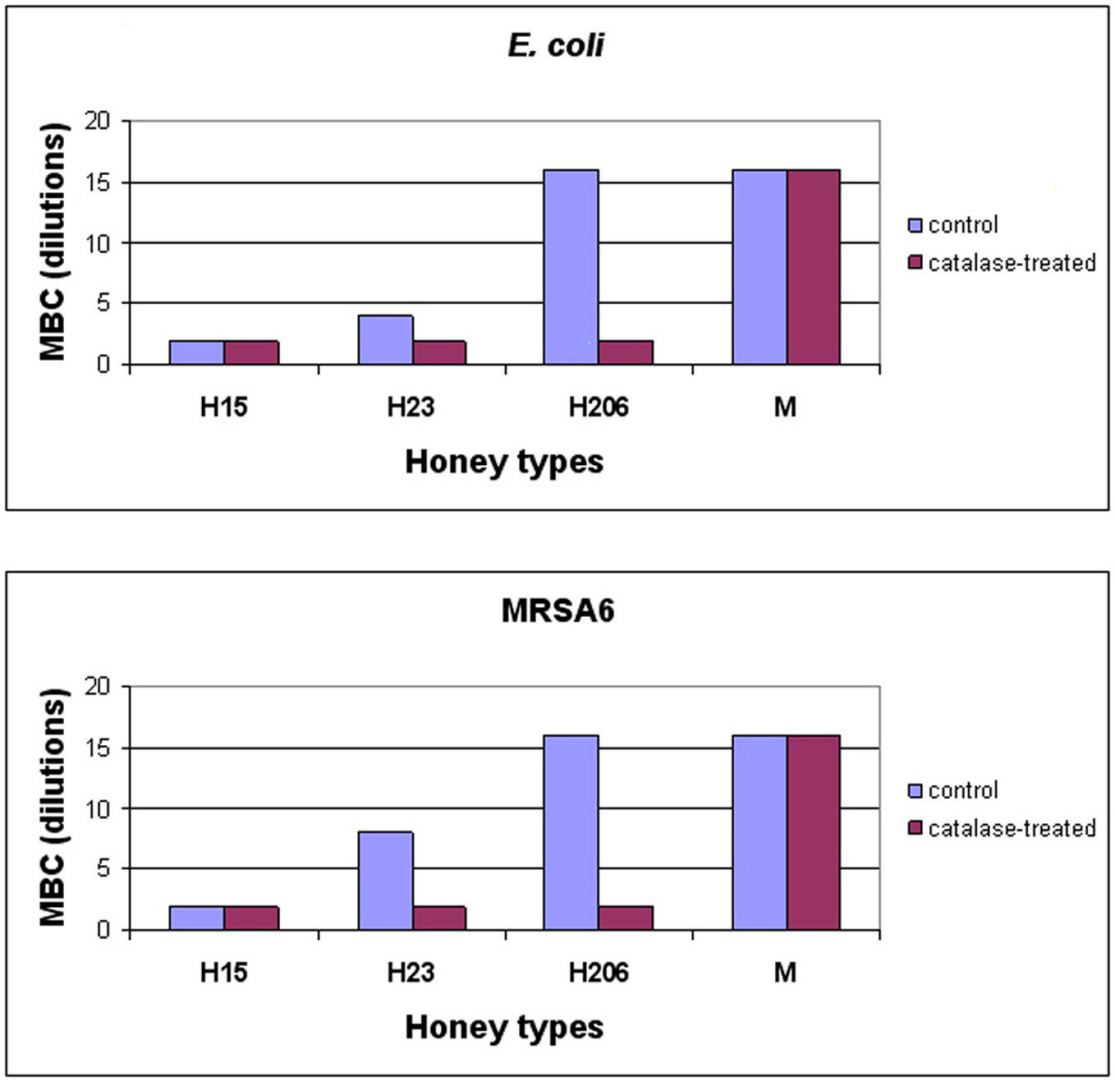
**Effect of honey's H_2_O_2_ on the survival of *E. coli* and MRSA6.** Control, untreated and catalase-treated honeys were incubated with *E. coli* and MRSA6 (10^7^ CFU/ml) at dilutions ranging from 2× (50% v/v) to 16× (6.25% v/v). The bacterial survival was analyzed by agar plating and colony enumeration. Values represent averages of three independent determinations where no viable colony counts were found on the agar plates. Conversion of dilution to honey concentrations (% v/v) is as follows: Dilutions:   2×  4×   8×       16×    32× Concentrations:   50%   25%   12.5%   6.25%   3.125%

To investigate whether H_2_O_2_ also influenced honey's bactericidal action, honeys were pre-treated with catalase (1000 U/ml honey) prior to the incubation with *E. coli* and MRSA6 and their MIC and MBC values were evaluated using broth microdilution assay followed by agar plating and colony enumeration, respectively. The removal of H_2_O_2_ reduced bactericidal effect of buckwheat honey H23 as evident by the decrease of the MBC value from 25% v/v to 50% (honey dilutions 4×–2×) against *E. coli* and from 12.5% v/v to 50% v/v against MRSA6 (honey dilutions 8×–2×) (Figure 3). The MBC of freshly obtained buckwheat honey H206 was decreased 8-fold from 6.25% v/v to 50% v/v (honey dilution 16×–2×) after H_2_O_2_ removal. Bactericidal activity of manuka honey and blueberry honey were unaffected by catalase treatment (Figure 3). Taken together, these observations imply that bacterial killing by buckwheat honeys required hydrogen peroxide.

### Detection and quantitation of hydroxyl radicals

**Figure 4:**
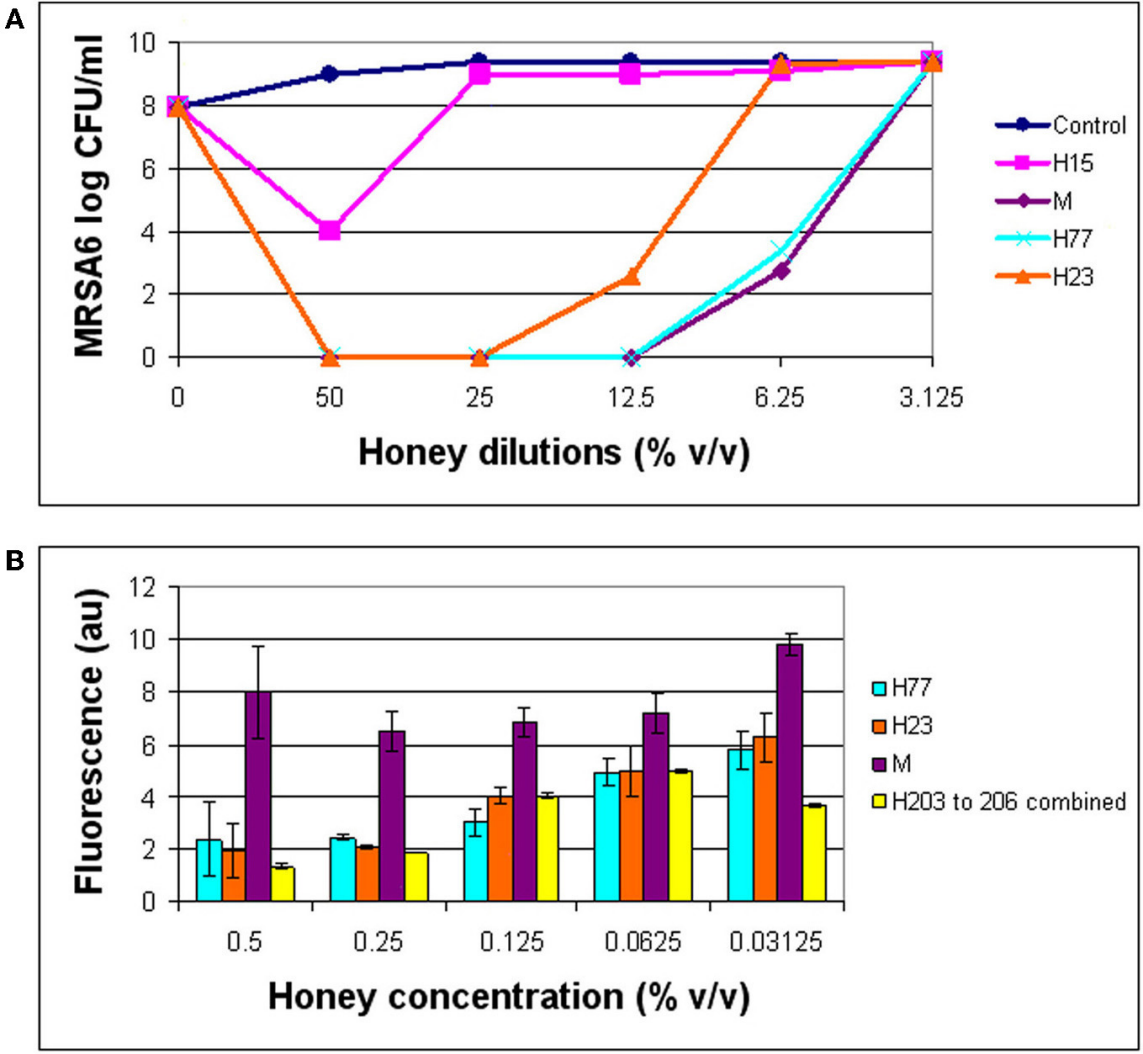
**Comparison of bactericidal effect of honeys in relation to the hydroxyl radical levels.** The growth inhibition, bactericidal effect of honeys on MRSA6, and hydroxyl radical generation were simultaneously analyzed using broth microdilution assay in the presence of aminophenyl fluorescein (APF) as ·OH trap. **(A)** Honey concentrations at which a complete eradication of MRSA6 was observed. **(B)** Comparison of ·OH radicals formed at bactericidal concentration of honeys (MBC) and beyond MBC.

The combination of 3′-(*p*-aminophenyl) fluorescein (APF) method with microdilution assay and colony enumeration by agar plating allowed us to monitor simultaneously the hydroxyl radical generation (by fluorescence), growth inhibition (by absorbance) and killing rates, respectively. As shown in (Figure 4), hydroxyl radical formation was dependent on the honey concentration/dilution. At lethal honey concentrations, ranging from 50% to 12.5% v/v, the levels of ·OH produced by different honeys were significantly lower than ·OH levels produced at non-bactericidal concentrations (12.5–3.125% v/v) (*t*-test, *p* < 0.0001). At maximum killing rate where no visible cells were detected on agar plates, the amount of radicals was 2–3 times lower than at MIC (Figure 4). In contrast, at honey concentrations beyond the MBC (12.5–3.125% v/v), the bacterial growth was observed on agar plates and ·OH radicals were clearly detected. These results indicated that the ·OH formation was associated with living cells while the dead cells did not accumulate appreciable levels ·OH.

## Discussion

A true therapeutic potential of honey as antibacterial agent depends on honey ability to eradicate infecting pathogens. In this study we have shown that honeys of buckwheat origin exhibited powerful bactericidal effect against standard bacteria as well as against MRSA and VRE. Honey ability to kill bacteria depended on (a) honey concentration, (b) inoculum size, and (c) the presence of H_2_O_2_. The MBC/MIC ratio ≤4 and the killing curves demonstrated that honeys at their MBCs caused >6 log_10_ CFU/ml reduction of colony counts, equivalent to complete bacterial eradication. While honey concentration above MIC were lethal (50–6.25% v/v), concentrations at and below MIC caused a rapid bacterial re-growth. This indicates that the narrow concentration range, in which honey was bactericidal, might have a significant impact on therapeutic outcomes. A slight change in the honey concentration or bacterial load would reduce or abolish its bactericidal effect. Therefore, the assessment of concentration-depended killing and the ratio of concentration to bacterial load turn out to be an important indicator predicting bactericidal effect *in vivo*. Often, the infected sites contain bacteria at a higher density than that used in our assays (10^7^ CFU/ml). It became evident that honey concentrations should exceed the MBC levels to ensure that the infecting organism is killed.

In this context, potency of honey's bactericidal activity emerged as another factor that influences bactericidal effect. We have shown here that bactericidal potency of honeys depended of honey variety and bacterial susceptibility to honey. The range of concentrations at which honeys exerted the maximal bactericidal effect was the broadest for manuka and buckwheat honey H77 (50–6.25% v/v), while blueberry honey showed bactericidal effect only at the highest concentration tested (50% v/v). Due to their potency, buckwheat honeys and manuka could be diluted up to 12.5% v/v and still maintained their maximal bactericidal efficacy. This leaves a space for an increase in honey dosage if the bacterial load exceeds that used in our experiments (10^7^CFU/ml).

No significant differences in susceptibility to honeys were found between antibiotic-resistant clinical isolates of MRSA and VRE and standard *E. coli* and *B. subiltis* bacteria, which suggests that honeys indiscriminately affects broad spectrum of microorganisms. These results are in agreement with previous findings obtained on honeys of different botanical and geographical origins (Cooper et al., 2000, 2002b; Lusby et al., 2005; Blair et al., 2009; Tan et al., 2009; Sherlock et al., 2010).

Honey effectively targeted both rapidly multiplying/dividing bacteria such as MRSA6 as well as those characterized by slower growth such as VRE2. The appropriate honey concentration in each case was required for killing. Honey concentration–dependent killing resembled a class of antibiotics whose activity is concentration dependent: aminoglycosides and quinolones. At low concentration, a primary mode of action of aminoglycosides is the inhibition of protein synthesis through an irreversible binding to the 30S ribosomal subunit. Quinolones, on the other hand, act by promoting cleavage of bacterial DNA in the DNA-DNA gyrase and type IV topoisomerase complexes, thereby inhibiting DNA synthesis and its repair. Recent studies showed however, that both groups of antibiotics have bactericidal activities at high concentrations; aminoglycosides—by creating fissures in the outer cell membrane (Montie and Patamasucon, 1995) and quinolones—by generating irreparable double-strand breaks in DNA resulting in rapid bacterial death (Hooper and Wolfson, 1993).

Whether or not honey action presents such a dual-mode concentration–dependent killing requires further investigations. Nevertheless, some advances have been made in this study toward this goal. Firstly, the causal relationship was observed between the concentration-dependent cell-kill on agar plates and DNA degradation. Honeys at their MBC caused extensive double-strand DNA breaks that led to a complete break down of genomic DNA. In contrast, honey at concentrations beyond the MBC did not degrade DNA. Thus, the complete DNA degradation appeared only at bactericidal concentration. This could suggest a bimodal effect of honey concentration on DNA integrity. Together, these data emphasize that DNA degradation, bactericidal effect and honey concentrations are interrelated. Secondly, we have found that hydrogen peroxide was also involved in the killing mechanism. Removal of H_2_O_2_ by catalase abolished honey bactericidal action. These results indicate that honey concentration and H_2_O_2_ both play active part in honey killing mechanism.

Earlier, we have shown that oxidative damage to bacterial cells was conferred by a coupling chemistry between H_2_O_2_ and honey polyphenols. The extent of oxidative damage depended on H_2_O_2_ levels and redox capacity of honey polyphenols (Brudzynski et al., 2012). These observations led us to the assumption that hydroxyl radicals generated from H_2_O_2_ via the polyphenol-mediated, metal-catalyzed Fenton reaction may be responsible for the observed bacterial growth inhibition and DNA degradation. In support of this notion was the fact that supplementation of honey with either Cu(II) or H_2_O_2_ resulted in a marked increase in bacteriostatic activity as indicated by over 30-fold decreased in MIC_90_ (from 6.25% v/v to less than 0.78% v/v in a case of Cu(II) supplementation) (Brudzynski and Lannigan, 2012). Subsequently, by including APF as ·OH trap in our broth microdilution assay, we have demonstrated a direct relationship between the ·OH generation and growth inhibition of MRSA and VRE.

However, in this study, we could not conclusively identify ·OH radicals as inducers of bacterial cell death. We did not find the quantitative relationship between ·OH levels and honey bactericidal effect. It could be argued that ·OH radicals were effectively scavenged by honey or bacterial antioxidants and hence not detectable by APF. However, neither honey nor bacterial ·OH scavengers were able to protect bacterial cells against the lethal oxidative damage. Rather, we are tempted to assume that both DNA degradation and the abrupt destruction of bacterial cells represent ·OH footprint, the direct or indirect consequence of fast acting ·OH radicals. The unpaired electron of the hydroxyl radical is highly reactive but short-lived species. Its reaction rates exceed 10^9^ M^−1^ sec^−1^ in biological systems (Roots and Okada, 1975). Thus, the action ·OH radical could be only examined indirectly by the detection of the lasting effects of its toxic action. Our data indicated that the generation of ·OH radicals was significantly correlated with living but not dead cells (*p* < 0.0001). The cell death and extensive degradation of genomic DNA may therefore represent this lasting footprint of ·OH radical action. It has to be mentioned here that DNA degradation is a widely accepted marker of oxidative damage caused by ·OH radical action in a variety of model systems (Rozenberg-Arska et al., 1983; Mello Filho and Meneghini, 1984; Imlay and Linn, 1988; Sahu and Gray, 1993; Yang et al., 2006; Keyhani et al., 2007).

Although the role of hydroxyl radicals have been increasingly recognized as the most significant contributor to cell death among reactive oxygen species (Gutteridge et al., 1998; Imlay, 2003; Kohanski et al., 2007, 2010), it is possible that other free radicals generated from the initial Fenton reaction may be the actual effector molecules. Further studies on the role and significance of ·OH radicals in bactericidal activity of honey are needed to reconcile its action with the concentration-dependent killing.

### Conflict of interest statement

The authors declare that the research was conducted in the absence of any commercial or financial relationships that could be construed as a potential conflict of interest.
